# The Zebrafish GenomeWiki: a crowdsourcing approach to connect the long tail for zebrafish gene annotation

**DOI:** 10.1093/database/bau011

**Published:** 2014-02-26

**Authors:** Meghna Singh, Deeksha Bhartiya, Jayant Maini, Meenakshi Sharma, Angom Ramcharan Singh, Subburaj Kadarkaraisamy, Rajiv Rana, Ankit Sabharwal, Srishti Nanda, Aravindhakshan Ramachandran, Ashish Mittal, Shruti Kapoor, Paras Sehgal, Zainab Asad, Kriti Kaushik, Shamsudheen Karuthedath Vellarikkal, Divya Jagga, Muthulakshmi Muthuswami, Rajendra K. Chauhan, Elvin Leonard, Ruby Priyadarshini, Mahantappa Halimani, Sunny Malhotra, Ashok Patowary, Harinder Vishwakarma, Prateek Joshi, Vivek Bhardwaj, Arijit Bhaumik, Bharat Bhatt, Aamod Jha, Aalok Kumar, Prerna Budakoti, Mukesh Kumar Lalwani, Rajeshwari Meli, Saakshi Jalali, Kandarp Joshi, Koustav Pal, Heena Dhiman, Saurabh V. Laddha, Vaibhav Jadhav, Naresh Singh, Vikas Pandey, Chetana Sachidanandan, Stephen C. Ekker, Eric W. Klee, Vinod Scaria, Sridhar Sivasubbu

**Affiliations:** ^1^CSIR Institute of Genomics and Integrative Biology (CSIR-IGIB), Mall Road, Delhi 110007, India, ^2^Academy of Scientific and Innovative Research (AcSIR), Anusandhan Bhawan, Delhi 110001, India, ^3^Acharya Narendra Dev College, Delhi University, Govindpuri, Kalkaji, New Delhi 110019, India, ^4^Dr. B. R. Ambedkar Center for Biomedical Research, University of Delhi, Delhi 110007, India, ^5^Department of Genetics, University of Delhi South Campus, Benito Juarez Road, Dhaula Kuan, New Delhi 110021, India and ^6^Mayo Clinic, Rochester, MN, USA

## Abstract

A large repertoire of gene-centric data has been generated in the field of zebrafish biology. Although the bulk of these data are available in the public domain, most of them are not readily accessible or available in nonstandard formats. One major challenge is to unify and integrate these widely scattered data sources. We tested the hypothesis that active community participation could be a viable option to address this challenge. We present here our approach to create standards for assimilation and sharing of information and a system of open standards for database intercommunication. We have attempted to address this challenge by creating a community-centric solution for zebrafish gene annotation. The Zebrafish GenomeWiki is a ‘wiki’-based resource, which aims to provide an altruistic shared environment for collective annotation of the zebrafish genes. The Zebrafish GenomeWiki has features that enable users to comment, annotate, edit and rate this gene-centric information. The credits for contributions can be tracked through a transparent microattribution system. In contrast to other wikis, the Zebrafish GenomeWiki is a ‘structured wiki’ or rather a ‘semantic wiki’. The Zebrafish GenomeWiki implements a semantically linked data structure, which in the future would be amenable to semantic search.

**Database URL:** http://genome.igib.res.in/twiki

## Introduction

Recent advances in genomics have required biologists to revisit accepted paradigms of how genes function and interact, as well as how genes cooperate to modulate a diverse array of complex biological processes such as development, metabolism and behavior (1). Application of advanced genomic approaches in humans and model organisms, including worms, flies, fish and rodents, has generated vast amounts of data, which has been reported in publications and public databases (2–4). Such publications typically describe salient features and general patterns of the genome-wide data, while the frequently large and multidimensional experimental data sets are presented as supplementary information or databases. Furthermore, the data generation process spans multiple laboratories involving diverse techniques, adding to the complexity of data presentation. All these factors create barriers to data integration including incompatible file formats and improper semantics. Consequently, this collective wealth of genomic and functional information remains isolated with little space for downstream integrative analysis to advance biological understanding.

Integration of these data sets into a common platform has been inhibited by the need for systematic manual curation (5–8) of information from unstructured data sources (published articles and supplementary literature) and from structured entities (databases and other structured data sets). The massive volumes of dynamic bioinformatics data pose serious challenges to biocurators. On one hand, the sheer volumes of data make it impossible for a single individual to connect the dots; on the other hand, the dynamic (and sometimes volatile) information makes it nearly impossible to create spatial and temporal snapshots of gene products and their functions across an entire genome. Despite these significant challenges, the literature abounds with models and examples of successful integration of resources and manual curation via community participation (9, 10). Perhaps the best-known example of a successful community-based curation model is the ‘wiki’ solution proposed by Ward Cunningham, which was systematically taken up by the general internet user community to create the large common knowledge repository Wikipedia (11).

Such community participation in an open environment has been successfully applied for covering the long tail in biological annotations (12–15). These methods have also been tested for genomic applications. The term ‘long tail’ was popularized by Chris Anderson (16) to describe the retailing strategy of selling small quantities of large number of unique goods versus the large sale of fewer popular goods. This term has been used in science to describe how people tend to access the articles in popular journals and miss the important data found in less popular journals or buried deep in the supplementary material of the manuscripts. So, in science, the ‘long tail’ is about collecting and connecting these missing links. In recent years, wiki-based annotation platforms, such as WikiProteins (17), WikiPathways (18) and WikiGenes (19), have enjoyed broad community participation. WikiProteins is a semantic web-based (20–22) portal modeled on wiki pages with connected knowlets of >1 million biomedical concepts. There has been a general trend toward using wikis as collaborative tools due to their simplicity and ease of use (8, 9). One major limitation of wikis as biocuration platforms is that the relevant data are inherently unstructured, organized in sentences and paragraphs, hindering text-mining and integrative analysis by machine logic. Structured wikis (20, 22) have been proposed to address this problem and allow an easy processing and sharing of the data by the users. Structured wikis allow creation of relations between different data sets or data points using standard ontologies enabling machine-readable links and easing the integration and analysis of large data sets.

Zebrafish (*Danio rerio*), a popular vertebrate model organism, has a genome of ∼1.5 billion base pairs distributed over 25 chromosomes. The zebrafish reference genome (http://www.ensembl.org/Danio_rerio/Info/Index) and genes and transcript annotations are readily available from major genome browsers and databases such as National Center for Biotechnology Information (http://www.ncbi.nlm.nih.gov/genome/guide/zebrafish), Ensembl (23), University of California Santa Cruz (UCSC) genome browser (24) and Zebrafish Model Organism (ZFIN) databases (25). Many post-genomic data sets, including the complete genome of a wild zebrafish (26), Expressed Sequence Tags (EST) and cDNA collections; transcriptome and genome variations; as well as a host of related information including gene loci, primary transcript and alternatively processed transcripts and protein information are also available for this excellent model system. Specialized resources such as the Zebrafish Mutant Collection (27), the Zebrafish Mutation Project (http://www.sanger.ac.uk/Projects/D_rerio/zmp/), Zebrafish Tilling Project (https://webapps.fhcrc.org/science/tilling/), zinc finger nuclease-targeted mutations (28), zTrap (29), ZETRAP 2.0 (30) and Zfishbook (31) (http://zfishbook.org/) provide periodic updates regarding the transgenic and mutant lines that have been generated. Previously, we created the FishMap (32, 33) database to integrate the genome-scale information on zebrafish into a centralized data repository with a visual interface. This platform combines computational predictions with experimental data sets, and is equipped with interfaces for visualization of zebrafish genome-scale data and integrative analysis and is widely used by the zebrafish scientific community.

We have applied the structured wiki concept to create a semantically organized system for community curation (4) of gene function in zebrafish. We involved community participation to manually and systematically curate information from published literature on gene and gene functions into a structured annotation portal. The community members were given due credit for their contributions using a microattribution system, which was later converted into an authorship in this manuscript (34, 35). The system is akin to a wiki in many ways, albeit with a structured format, which would allow for semantic integration of content and serve as a structured interface for aggregating and storing information. The resource holds synchronized and updated annotations for a large number of zebrafish genes. This information is continuously enriched by collective community inputs and is a model for community involvement for biocuration in genomics for model organisms.

## Materials and Methods

### Annotation protocol

**Figure 1.**
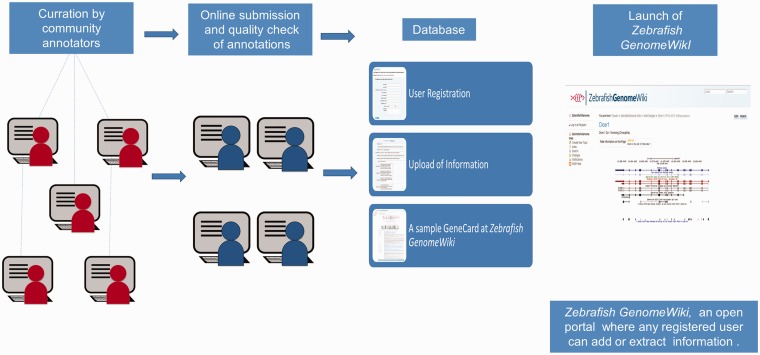
A schematic representation of the annotation protocol. The annotators were given a set of genes and a ready reference. The annotated entries were submitted through online media and were quality checked before upload.

We used a community curation approach. Each volunteer went through an initial training process under the guidance of a curator, during which the volunteer was familiarized with the concepts of annotation, standards and databases and how to systematically assemble annotation information for a gene. We followed a standard gene annotation protocol, which included literature survey and checking for information in biological databases. After the volunteers were introduced to the process and standard protocols, they were provided with a template GeneCard and asked to annotate the genes using information as described in the published articles and corresponding databases (Supplementary File S2). The annotation process began with selecting an available gene of interest and systematically collecting information for a particular gene, including the gene name, gene identifier (ID), RefSeq ID and transcript IDs, in a preapproved format provided in the Zebrafish GenomeWiki. The key reference anchor points in the Zebrafish GenomeWiki for any gene is the gene name, gene ID and RefSeq ID. Therefore, we ensured that key reference anchor IDs correlated between the ZFIN, Ensembl and Zebrafish GenomeWiki databases. The annotation process also included extensive literature survey for both biological functions and mutant phenotypes. The volunteers were encouraged to discuss and share information online through online media. Curators further manually cross-checked all entries for inadvertent annotation errors (Figure 1). A self-explanatory tutorial provides a quick user reference guide for the new user, which is also provided as a link in the Zebrafish GenomeWiki web page (Supplementary File S1).

### Zebrafish gene nomenclature

We used the current zebrafish reference genome from UCSC/Ensembl (Zv9 build) for our curation. For gene nomenclature, we used the official nomenclature from ZFIN. For each annotated gene, the ZFIN-approved gene name anchors its corresponding GeneCard (36) in the Zebrafish GenomeWiki. We have provided links and alternate nomenclatures available at other databases for ready comparison. Wherever alternate or redundant gene names have been used, we have also retained them for reference.

### Organization of information webs and subwebs

Zebrafish GenomeWiki is a structured wiki, hierarchically organized into webs and subwebs. The wiki follows a hierarchy wherein the main web pages are the gene pages providing gene level information and links to other webs and external resources. The wiki also incorporates FishNet, a search engine for zebrafish gene annotation and resources. FishNet uses Swish-e (Simple Web Indexing System for Humans—Enhanced) (http://swish-e.org/) for efficient and speedy retrieval of the data. It indexes the entire data and quickly extracts the data on the basis of the keyword provided.

### Data formatting and exchange

Information from the various sources is converted into a uniform standard format as specified in the Zebrafish GenomeWiki template. The primary entry of each gene is a template, called the GeneCard (36). Each entry on the GeneCard or any of the annotation templates in the subwebs have defined standard input formats, which enable interlinking between webs and subwebs, and external databases. The complete list of ontologies used in the webs is summarized in Supplementary Table S1. In Zebrafish GenomeWiki, a registered user can edit as well as extract information. Any changes made to a particular web page can be tracked in web history, allowing a check against any kind of mismatch or obsolete information upload.

## Results and Discussion

### Zebrafish GenomeWiki components

**Figure 2.**
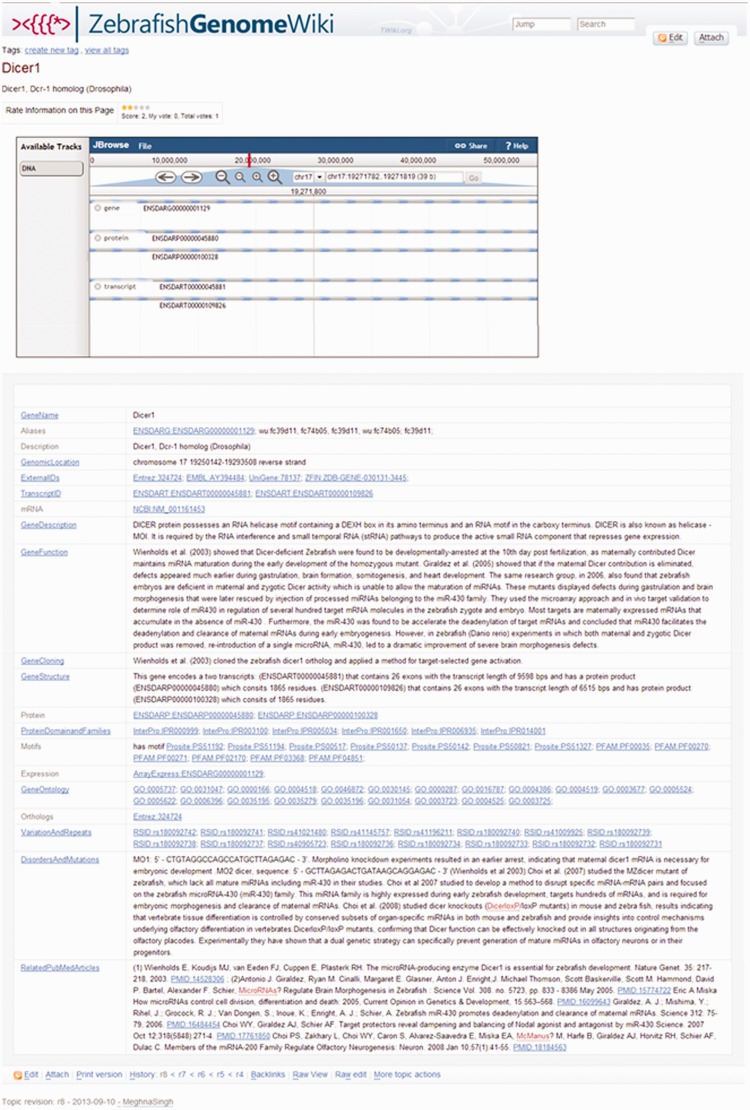
Screenshot of a sample GeneCard entry. In the Zebrafish GenomeWiki, the top panel of the page consists of a genome browser interface. The GeneCard is divided into a number of fields each of which consists of information for a particular gene. All the entries highlighted in blue in the information panel are the linkouts to the respective source databases. The bottom panel of the page provides the information about the revision history of the particular gene including an option to edit the page. The information about the last annotation and the annotator is available at the bottom left corner of every page.

The Zebrafish GenomeWiki provides a seamless search interface through FishNet, a multi-resource search engine for zebrafish gene annotation. The FishNet query page in turn provides links to the corresponding GeneCard. The main GeneCard page is categorized into a number of subwebs. The subwebs attempt to cover the majority of important data related to zebrafish genomics, including but not limited to human orthologs present in zebrafish, mutants, morpholinos, diseases models, transposon insertion sites and information on noncoding RNAs (Figure 2). Each gene ID is hyperlinked to the main GeneCard entry. A GeneCard provides a vivid explanation of the gene. It consists of a number of entry points such as the Gene ID, GeneName, Transcript ID, Protein ID, Refseq ID, known GeneFunction, GeneOntology, GeneExpression and references. The reference section provides direct links to the related publications from where the information has been extracted. The highlighted entry under each category links to a new web page containing related information and has semantic linkouts wherever necessary. Each GeneCard page also provides links to external web pages. The Zebrafish GenomeWiki is also provided with a sandbox feature that guides the user on how to create new topics.

### Annotating genes in the Zebrafish GenomeWiki

In the present version of Zebrafish GenomeWiki, ∼40 students and curators including 30 annotators from eight different laboratories and universities were involved. These students were involved in initial curation of ∼600 genes and then further the quality check and upgradation of the database (Supplementary Figure S1). The success of community annotation (4, 37) primarily lies in the availability of standard operating protocol or format for data organization and easy retrieval, a centralized annotation submission and an expert quality check to avoid any ambiguity or duplication. Furthermore, the data points should link back to each other to establish a proper workflow. The Zebrafish GenomeWiki follows a uniform template on which the information is collected and curated. Existing genes already have a ready template page in the Zebrafish GenomeWiki, and new gene templates can be generated as and when required. Key reference anchor points such as the ‘GeneName’, ‘Gene ID’ and ‘RefSeq ID’ provide an entry point for the annotation in the Zebrafish GenomeWiki. Biological information collected through extensive manual review of literature and unpublished information from individual laboratory web pages or databases are centralized and then undergo substantial quality checks for accuracy and precision. Finally, the curated data are collated into individual GeneCards in the Zebrafish GenomeWiki.

### Correcting annotation mistakes and database maintenance in the Zebrafish GenomeWiki

The Zebrafish GenomeWiki provides a dynamic real-time interface for uploading and extracting information. It saves information regarding all updates and modifications performed by any user in real time. Therefore, if a user inadvertently introduces a mistake during the annotation process, the error can be easily modified by reverting back to the last correct version (see Supplementary File S1 for details). The database also permits users to download a database snapshot for archiving on their private servers.

Additionally, the complete database is periodically (quarterly) archived for security purposes. and the database is updated quarterly. The data are derived from ensemble and ZFIN and is thereon updated automatically.

### Microattribution—ensuring credits for the contributors

Success of any wiki-based resource depends on active community participation. Because studies show that community members are most willing to contribute voluntarily when their contributions are readily recognized (34, 37), the Zebrafish GenomeWiki follows a microattribution system of credit sharing. Both correct and incorrect annotation contributions are tagged with the respective user’s unique ID. This ensures that credits for all the contributions go back to the user in real time. The microattribution information for the individual contributions is provided as a subweb for ready reference. The microattribution system linked to contributions is also used to ensure proper credit sharing. For example, users who have contributed to the contents of the current version of Zebrafish GenomeWiki database have been listed as coauthors in this manuscript based on their microattribution credits.

### Current status and the way forward for Zebrafish GenomeWiki

In the first version of the Zebrafish GenomeWiki, we have manually curated 600 genes for which the users have provided biological annotations. In addition, the Zebrafish GenomeWiki also contains ∼52 896 transcripts and 4150 proteins. The Zebrafish GenomeWiki, we describe here, is a starting point toward systematically collating annotations for genes and gene products within a structured wiki platform. The system solves to a large extent the issues with unstructured data on wiki-based annotation platforms and provides for an alternative strategy combining the advantages of a structured database-driven annotation system with the openness of a wiki-based annotation system. The future would be to integrate this into standard ontologies and make available as Resource Description Framework (RDF) tuples, thus making it compatible with the semantic web technology (21). Recent technologies have enabled us to convert database formats to RDF tuples, and many visualization and search strategies using RDF data are just emerging. We hope this would significantly enrich ongoing projects, such as the Linked Data Initiative (http://linkeddata.org/).

We understand that to encourage and sustain high-quality annotation activity, apart from the peer review system and organizational hierarchy, there should be enough incentives to sustain the endeavor. All open source and open data initiatives have inbuilt incentive mechanisms, which sustain the organization and the community. We would be keen to work with journals and databases, and with upcoming initiatives like the Bioresource Research Impact Factor (38) and make the resource, annotations and annotator information interoperable. In future, the training manuals including annotation and curation protocols will be made available on the database to improve the quality of the data sets uploaded and minimize spurious information. We encourage all zebrafish research community members to actively participate in this collaborative environment for connecting the long tail for zebrafish genome annotation.

## Supplementary Data

Supplementary data are available at *Database* Online.
